# Reduction in corpora lutea number in obese melanocortin-4-receptor-deficient mice

**DOI:** 10.1186/1477-7827-7-24

**Published:** 2009-03-24

**Authors:** Mara Sandrock, Angela Schulz, Claudia Merkwitz, Torsten Schöneberg, Katharina Spanel-Borowski, Albert Ricken

**Affiliations:** 1 Institute of Anatomy, Medical Faculty, University of Leipzig, 04103 Leipzig, Germany; 2 Institute of Biochemistry, Medical Faculty, University of Leipzig, 04103 Leipzig, Germany

## Abstract

Obese melanocortin-4-receptor-deficient (MC4R-/-) male mice are reported to have erectile dysfunction, while homozygous MC4R-/- female mice are apparently fertile. A recently established obese mouse strain, carrying an inactivating mutation in the MC4R gene, revealed difficulties in breeding for the homozygous female mice. This prompted us to determine the presence of follicles and corpora lutea (CL) in ovaries of MC4R-/- mice aged 3–6 months in comparison to wild type (MC4R+/+) littermates. Serial sections of formaldehyde-fixed ovaries of mice with vaginal signs of estrus and metestrus were assessed for the number of healthy and regressing follicles and CL. The number of CL, as an estimate for the ovulation rate, decreased to zero during aging in MC4R-/- mice. The number of small- (diameter 100–200 micrometer) and large-sized follicles namely antral follicles (diameter >200 micrometer) were slightly increased in MC4R-/- compared to MC4R+/+ mice. Greater differences were found in very large to cystic follicles, which were more numerous in MC4R-/- mice. The number of regressing antral follicles was higher in the MC4R-/- group compared to the MC4R+/+ group. This was associated with a wide range in the number of collapsed zonae pellucidae as the last remnants of regressed follicles. A conspicuous hypertrophy of the interstitial cells was noted in 6-month-old MC4R-/- mice. In conclusion, cystic follicles and the reduction in CL number point to a decreased ovulation rate in obese MC4R-/- mice.

## Background

Obesity has become a major health problem affecting more than a quarter of all adults in countries with high living standard [1]. Due to the increasing impact on public health care, research on the metabolic control of body weight, food intake and energy expenditure is required. The development of obesity depends on multiple factors including food intake, body exercise, but also on inherited variants and defects in the endocrine regulation cycle of energy homoeostasis [2,3]. Defects in the genes for leptin, leptin receptor, proconvertase 1, pro-opiomelanocortin (POMC) and the melanocortin receptors MC3R and MC4R have been associated with obesity [4-6]. Dysfunction of the MC4R appears to be a relatively common factor in early onset obesity and more than 80 different obesity-associated MC4R mutations have been identified so far [7].

Hypertension, insulin resistance and diabetes mellitus but also reduced fertility are often associated with obesity in humans [8,9] and also in mice [10,11]. Female ob/ob mice, lacking leptin, are infertile because of ovarian failure. Anovulation, low steroid hormone levels and a high rate of follicular atresia are characteristic for leptin deficiency. There is consent that the leptin system controls gonadotropin-releasing hormone (GnRH) secretion and, therefore, gonadotropin (follicle stimulating hormone/luteinizing hormone) production [10]. However, evidence for the involvement of the melanocortin/MC4R system in gonadotropin release and production is lacking.

Several MC4R-deficient mouse models have been developed, including mouse strains carrying gene deletion [12] or complete (Y302C) and partially (I194F) inactivating mutations [13]. The obese phenotype of male MC4R-deficient mice is further characterized by erectile dysfunction and changed sexual behavior [14]. When the male of the breeding pair has free access to a running wheel their fertility is restored [15]. This indicates that reduced fertility due to erectile dysfunction in male MC4R-/- mice is secondary to obesity. Reduced female fertility in MC4R-/- mice has not been described yet. However, we and others (M. Augustin, Ingenium Pharmaceuticals AG, Martinsried, Germany; personal communication) have observed that pregnancy of female MC4R-/- mice (Y302C and I194F strains) older than 3 months is rather rare. This observation encouraged us to investigate the morphology of the mice ovaries in detail. Our findings show that the reduced fertility in female MC4R-/- mice is associated with the reduction of CL and apearence of cystic follicles in 6-month-old MC4R-/- mice and indicates a reduced ovulation rate in MC4R-deficient mice.

## Methods

### Mouse strains

Using the chemical random mutagenesis technique with the germ line supermutagen N-ethyl-N-nitrosourea (ENU), a mouse model for a mutant G protein-coupled receptor (MC4R) was generated by Ingenium Pharmaceuticals AG, Martinsried, Germany. Functional *in vitro *analysis of the mouse MC4R containing the mutation I194F revealed a partial loss of receptor function (~40-fold reduced agonist potency). At the *in vivo *level this mutant causes the full obese phenotype as observed in a mouse strain containing a MC4R mutation (Y302C) with a complete loss-of-function in *in vitro *assays [13]. We used the I194F mouse strain and for simplicity, refer to the strain as MC4R-/-. Mice were bred and maintained under specific-pathogen-free conditions at the local animal care facility, where lights were automatically controlled (12 h light/12 h dark). Free access was given to chow and water. Mice were sacrificed by CO_2 _overdose. The treatments complied with the regulations issued by the local Animal Care Committee and with the regulations established by the German Council for Animal Care. The initial I194F C3HeB/FeJ (C3H) mouse strain [13] was crossed into the C57Bl6J (B6) background to reduce the C3H genetic influence on the number of offspring and estrous cyclicity, as C3H mice are more susceptible to irregular cyclicity than B6 mice [16]. Breeding was carried out with heterozygous breeding pairs. Genotyping of the littermates was performed by polymerase chain reaction (PCR) followed by *Bsp*HI (New England Biolabs, Frankfurt, Germany) restriction analysis. The following primers and PCR conditions were used: 5'-taccctgttaaacagtacggatac-3' (sense) and 5'-gaacatggaaatgaggcagatca-3' (antisense) creating a *Bsp*HI-site in MC4R+/+ sequence, conditions: 94°C 3 min; 35 cycles of 94°C 30 sec, 58°C 30 sec and 72°C 1 min. Products were digested with *Bsp*HI and fragments were separated in a 3% agarose gel.

We analysed data from two cohorts of female MC4R+/+ and MC4R-/- mice. The first cohort comprised animals with a mixed C3H × B6 background (3 to 6 crosses into B6). These animals were sacrificed with 3 to 6 months of age. The second cohort comprised animals with an essentially pure B6 background (at least 10 crosses into B6, table 1) with an age of 6 months.

**Table 1:** Number of animals according to age, genotype and vaginal histology.

		**Age (months)**				
		**3**	**4**	**5**	**6**	**6**
**Genotype**	**Estrous cycle**	number of animals				
MC4R+/+	estrus	1	2	1	3	4
	metestrus	3	2	2	2	9
MC4R-/-	estrus	2	4	3	4	10
	metestrus	2	0	2	0	3
		1^st ^cohort			2^nd ^cohort	

The genotype was identified by PCR analysis as described in *Methods*. Signs of estrous cycle stage were assigned retrospectively on the basis of vaginal histology. The 1^st ^cohort comprises animals at a mixed C3H/B6 background, the 2^nd ^cohort comprises animals at a B6 background.

The abdominal cavity was opened and the reproductive tract was uncovered and initially examined. The presence of fresh (hemorrhagic) and older (white opaque) CL was noted and the total number of surface CL was recorded. Thereafter, the reproductive tract was removed and immediately fixed with 4% formaldehyde in phosphate buffered saline (PBS), pH 7.2.

### Histology

Organs were fixed for at least 24 h and embedded in paraffin wax according to the commonly used histological technique. Paraffin sections of the vagina were stained with haematoxylin-eosin (HE), and changes of the vaginal epithelium characteristic of the estrous cycle stages were assessed [17,18]. Fresh and old corpora lutea at the initial macroscopically inspection indicated cyclicity and ovulations, but the number of CL appeared to be reduced in MC4R-/- mice compared to MC4R+/+ mice, focussing attention at the pre-and postovulatory periods. Therefore, only ovaries in the phase before ovulation (estrus) and the phase after ovulation (metestrus) were selected for serial sections (7 μm thick) along the longitudinal plane. Sections were collected into three alternate series of sections through the ovary at an interval of 28 μm (numbered 1a, 1b, 1c, 2a, 2b, 2c etc.). The 1^st ^series was stained with HE (numbered 1a, 2a, 3a etc). The 2^nd ^series was treated with the periodic-acid-Schiff (PAS) reaction [19] and the rest was kept as reserve series.

### Counting

The sections of the first cohort (24 animals with vaginal signs of estrus or metestrus under a mixed C3H × B6 background) were examined for morphological parameters to characterize ovarian follicular, luteal and interstitial cell development according to the literature [20]. Fresh and regressing CL from the present cycle and involuting CL from the previous cycles were counted in every 4^th ^section of an ovary (series of HE stained sections, every section on glass slide 1a, 2a, 3a etc.) by comparing the section with the preceding and following sections. The presence of CL of different stages in the same ovary made it possible to draw a relation between the different morphologies and age of formation [21]. Fresh CL were characterized by a still present central cavity, filled with blood and follicular fluid remnants or by prominent polyhedral to round luteal cells. Regression and involution of CL were recognizable by a general shrinkage of the luteal tissue, by vacuolated and scattered apoptotic cells (condensed chromatin, shrunken eosinophilic cytoplasm, and fragmentation into apoptotic bodies), by arterioles with thickened walls or by hyalinized material [22].

Follicles were also counted in every 4^th ^section of an ovary. Using an ocular scale the follicles were classified by diameter into small growing (100–200 μm), essentially preantral, large growing (200–400 μm), essentially antral and cystic follicles (>400 μm) [20]. To avoid double counting, only follicles with apparent nucleus of the oocyte (about 26 μm for mice oocytes [23]) were considered. Simultaneously with counting, the antral follicles were classified into healthy and atretic stages, following established morphological criteria were regression is defined by deformation and/or necrosis of the oocyte, more than 5% pyknotic granulosa cells and intercellular loosening of the granulosa cell layer [24]. The final number of follicles was calculated for a 1 mm thick ovarian section after the follicles of the whole ovary had been counted and assessed.

Collapsed zonae pellucidae were counted in 6 PAS-stained sections of an ovary at an interval of 168 μm (every first section on glass slide 1b, 2b, 3b etc.). The collapsed zonae pellucidae were considered as final stages of follicular atresia [20].

For the interstitial cells of the cortex, the number of nuclei was determined in a 100-μm^2 ^area with the help of an ocular grid. The areas had to be intact and free of prominent blood vessels. On average, one area per 5 HE-stained sections at an interval of 168 μm was evaluated. Here a total of 2 ovaries per mouse and a total of 4 mice per genotype at the age of 6 months were evaluated.

Based on the results of the morphological examination of the first cohort, we also examined 26 animals with vaginal signs of estrus or metestrus at B6 background (second cohort) exclusively for CL existence.

### Photographic documentation and statistics

Pictures were taken with a light microscope (Axioplan 2, Zeiss, Jena, Germany) equipped with a digital camera and the Image Access Software (Image, Glattburg, Switzerland). Data analysis was performed with Microsoft Excel 2003 updated for Box plot. Results were displayed as box-and-whisker plots showing the median, the "box" encircling the first (Q1) and third (Q3) inner quartiles, the "whiskers" presenting the farthest points (i.e., that are within 3/2 times the interquartile range of Q1 and Q3) and "crosses" the outliers of the data. Differences between MC4R-/- and MC4R+/+ mice were compared with Sigma Stat (Systat Software, Erkrath, Germany) using the Mann-Whitney Rank Sum Test. The results were considered as statistically significant when the p value was ≤ 0.05.

## Results

MC4R-/- mice partially or entirely crossed into B6 background were used to analyze an obvious reproductive deficiency. Obesity development of the present B6 crosses was similar as initially described for mice with a C3H/B6 50/50% background [13]. Ovary histology of female MC4R+/+ and MC4R-/- mice was characterized and quantified at different ages.

The presence of fresh and old corpora lutea at the initial inspection of the ovaries at laparatomy indicated cyclicity and ovulations in MC4R+/+ as well as MC4R-/- mice. Furthermore, characteristic changes of the vaginal epithelium (proliferation, mucification, keratinisation and desquamation) suggested existence of all estrous cycle stages independent of the receptor status and genetic background (not shown). Nevertheless, a stratified and cornified epithelium was strikingly more frequent among MC4R-/- mice (13 out of 17) compared to MC4R+/+ mice (7 out of 18).

**Figure 1:**
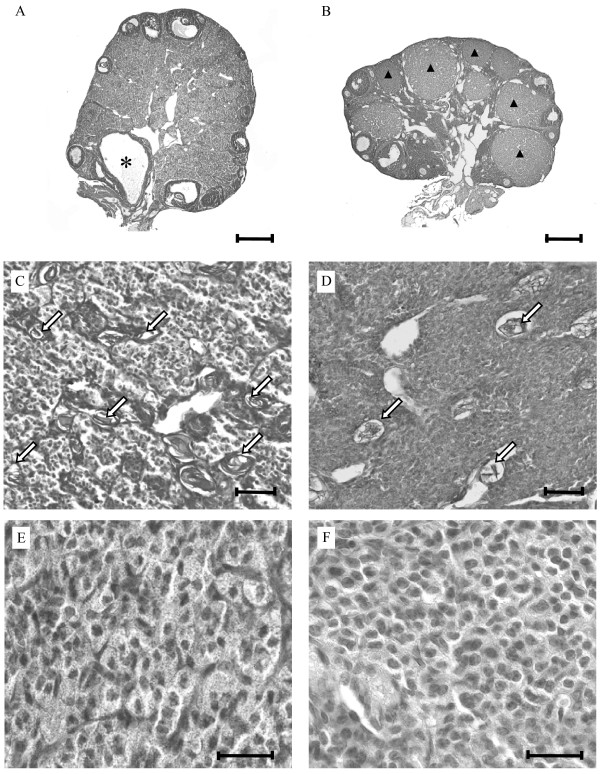
**Ovaries of 6-month-old MC4R-deficient mice differ in morphology compared to MC4R+/+ mice**. The figure shows representative ovary sections of mice with a mixed genetic C3H/B6 background. Under a B6 background, identical morphological changes were observed although less frequent. A and B: Ovaries of MC4R-/- mice (A) contain a cystic follicle (star) and no CL, while MC4R+/+ ovaries (B) show many CL (arrowheads). H.E. Scale bars: 200 μm. C and D: Collapsed zonae pellucidae as remnants of regressed follicles (arrows) are more obvious in MC4R-/- mice (C) compared to MC4R+/+ mice (D). PAS reaction. Scale bars: 50 μm. E and F: Interstitial gland cells are hypertrophied in MC4R-/- ovaries (E) compared to MC4R+/+ ovaries (F). H.E. Scale bars: 25 μm.

**Figure 2:**
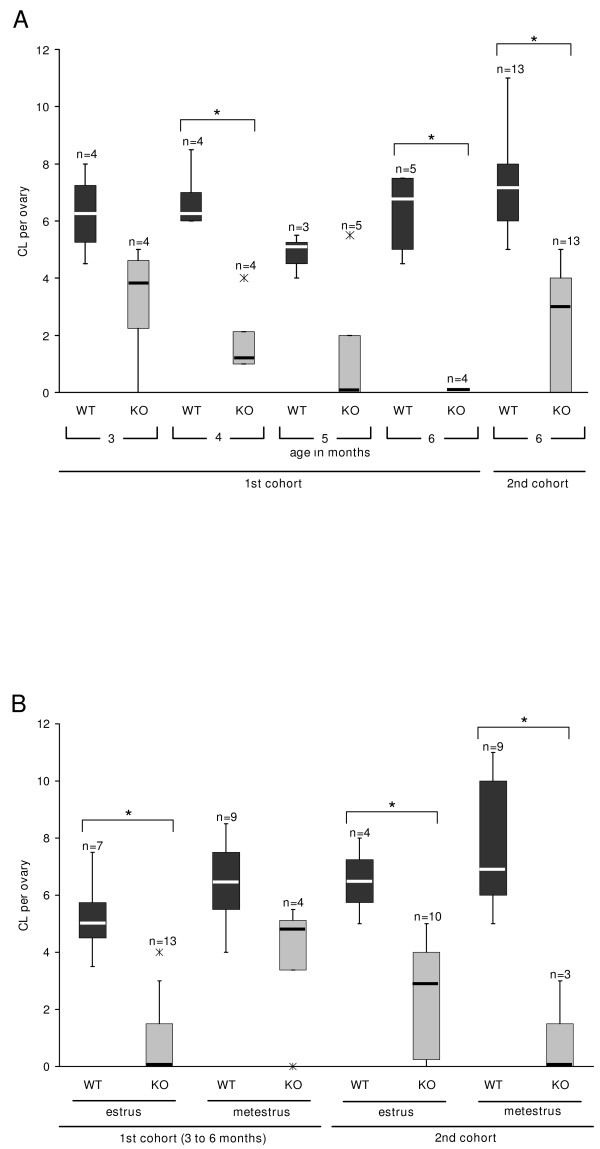
**Decrease in the number of CL in ovaries of MC4R-/- mice**. The number of CL was determined in completely dissected H.E. stained ovaries. Compared with MC4R+/+ mice the number of CL is significantly lower in MC4R-/- mice at a mixed C3H/B6 (1^st ^cohort) and at a B6 background (2^nd ^cohort). A: The difference in the 1^st ^cohort appears to increase with animal age in months. B: shows the data broken down into animals with vaginal signs of estrus and metestrus. Data are presented as box-and-whisker plots with median (line), interquartile range (box), minimum and maximum range (whisker) and outliers (cross); n indicates the number of animals; * indicates significant differences with p value ≤ 0.05.

Macroscopically, the ovaries of older MC4R-/- mice appeared to have fewer CL than MC4R+/+ mice. Therefore, we set out to histologically quantify this obvious difference in complete dissected ovaries of mice with vaginal signs of estrus and metestrus. The number of CL decreased in MC4R-/- mice between 3 to 6 months of age and were absent at the age of 6 months (Fig. 1A, Fig. 2A). In contrast, ovaries of corresponding 3- to 6-month-old MC4R+/+ littermates were found to consistently contain several CL of the present and/or previous estrous cycles (Fig. 1B). The differences in the CL number between MC4R-/- and MC4R+/+ mice were statistically significant for ovaries with vaginal signs of estrus (Fig. 2B).

**Figure 3:**
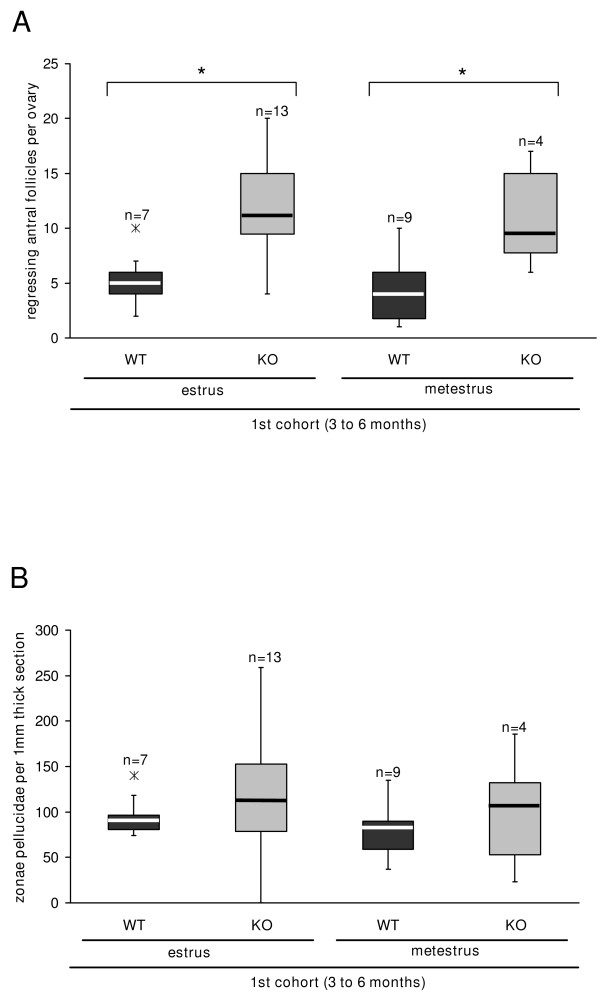
**Increase in the number of regressing antral follicles in ovaries of MC4R-/- mice**. The ovaries of MC4R-/- and MC4R+/+ mice at a mixed C3H/B6 background (1^st ^cohort) were serially sectioned and either H.E.-stained for counting regressing antral follicles in (A) or PAS-stained for counting zonae pellucidae in (B), the latter at an interval of 168 μm. The final number was calculated for either the whole ovary (A) or for a one millimeter thick ovarian section (B). A: Significantly more regressing antral follicles are counted in MC4R-/- mice compared to MC4R+/+ mice with vaginal signs of estrus and metestrus. B: The range in the number of collapsed zonae pellucidae is strikingly wider in MC4R-/- than in MC4R+/+ mice. Data are depicted as box-and-whisker plots with outliers; n indicates the number of animals; * highlights significant differences (p ≤ 0.05)

Examination of the follicles in ovaries of MC4R-/- and MC4R+/+ mice showed follicle types in preantral (small) and antral (large) stages of development (Fig. 1A, B, Table 2). It was noticed that in MC4R-/- ovaries, slightly more follicles of all sizes appeared and that larger follicles were often cystic (∅ > 400 μm) (Fig. 1A, Table 2), which likely indicated follicular atresia. Indeed, more regressing antral follicles were counted in ovaries of MC4R-/- mice (Fig. 3). This was associated with a wide range in the number of collapsed zonae pellucidae as follicular remnants (Fig. 1C, Fig. 3B).

**Table 2:** Number of intact and cystic follicles per ovary according to genotype, assigned vaginal signs of estrous cycle stage and follicle diameter.

	**Genotype**			
	MC4R+/+ (n = 7)	MC4R-/- (n = 13)	MC4R+/+ (n = 9)	MC4R-/- (n = 4)
**Follicle size in μm**	**estrus**		**metestrus**	
small (100–200)	**8.6 **(1–17)	**13.7 **(3–21)	**12.6 **(3–24)	**19.7 **(14–28)
large (>200)	**7.0 **(0–11)	**11.7 **(4–25)	**9.6 **(2–20)	**13 **(6–19)
cystic (>400)	**0.5 **(0–2)	**3.4 **(1–7)	**0.5 **(0–2)	**0.25 **(0–1)

Ovaries of 3 to 6 months old mice of the first cohort were serially sectioned along the longitudinal plane. Every 4^th ^section was analysed. Follicles were counted as described in *Methods*. Data are given as means. The numbers in parentheses indicate the range.

**Figure 4:**
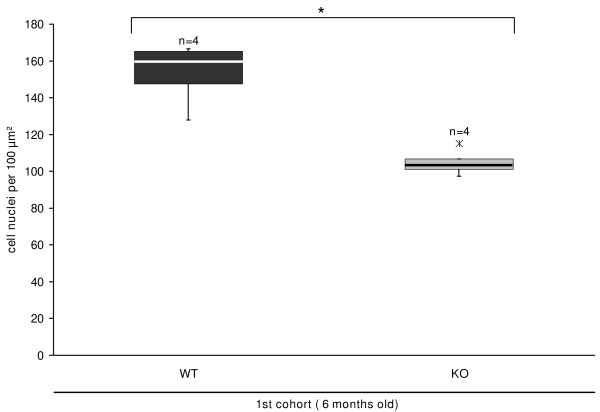
**Hypertrophy of interstitial gland cells in ovaries of MC4R-deficient mice as measurable decrease in nuclear density**. To determine whether hypertrophy of interstitial gland cells (Fig. 1E) occurs to a measurable extent, the nuclear density was determined per 100 μm^2^-sized areas of interstitial gland cells. Statistically significantly less nuclei are counted in 6-month-old MC4R-deficient C3H/B6 mice than in MC4R+/+ littermates. Data are presented as box-and-whisker plots with outliers; n indicates the number of mice; * highlights significant differences (p ≤ 0.05).

Additionally, the interstitial cortex in ovaries of 6-month-old MC4R-/- mice showed morphological differences compared to the MC4R+/+ mice. In MC4R-/- mice the cortex was occupied by clusters of hypertrophied epitheloid-like interstitial gland cells with clear appearance (Fig. 1E). The difference in interstitial cell size between the two groups was validated by counting the cell nuclei per defined area. On average, 105 cell nuclei in MC4R-/- compared to 157 cell nuclei in MC4R+/+ mice depicted a disparity of 34% (p < 0.05; Fig. 4).

The results described above were obtained with mice on a mixed genetic background (C3H/B6). For females of the C3H strain an irregular cyclicity is described [17]. To verify the results obtained with MC4R-/- mice on the mixed C3H/B6 background and to minimize the influence of the genetic background on the results, additional females were examined in a second cohort. Here, female MC4R-/- and MC4R+/+ mice with a B6 background (>10 crosses into B6 strain) were used. For 26 female mice with vaginal signs of estrus or metestrus the existence and number of CL was examined.

As found in the first cohort, CL were also reduced in MC4R-/- mice with a B6 background, although the reduction was less pronounced (Fig. 2A, 2B).

Additionally, a stratified and cornified vaginal epithelium was also more frequently in B6 MC4R-/- females (10 out of 13) compared to MC4R+/+ females (4 out of 13).

## Discussion

In this study, we examined the ovarian morphology of obese mice with an ENU-induced obesity-causing mutation (I194F) in MC4R. Two populations of mice differing in the strain background were used to reduce a strain-specific phenotype: a mixed C3H/B6 background (1^st ^cohort) and B6 background (2^nd ^cohort). In both cohorts the MC4R-/- mice showed morphological changes in the vaginal epithelium and in the ovary, which indicated that female MC4R-/- mice initially have regular ovarian cyclicity and fertility [4,10,25], which later on declines. This is in contrast to female B6 ob/ob mice, which are infertile throughout their life [26,27]. Mice become sexually mature after the 38^th ^day of life, thereafter undergoing a 3–6 day estrous cycle with stages of proestrus, estrus, metestrus and diestrus [28]. The regularity of the estrous cycle is under marked genetic influence. Beyond the age of 5 to 6 months B6 mice cycle regularly and are in the period of maximum cycle frequency [17,29]. C3H mice are at the same time more susceptible to disturbed cyclicity than B6 mice [16]. This strain difference could explain why the main morphological change in MC4R deficient ovaries reported here, the reduction in CL, is more pronounced in 6-month-old MC4R-/- mice at a mixed C3H/B6 background than in age-matched MC4R-/-mice at a B6 background.

The observed reduction in CL formation in MC4R-/- mice during aging (1^st ^cohort) together with the increased follicular atresia and frequent vaginal stratification and cornification indicates that the ovarian cycle, which normally persists up to the age of two years, could prematurely cease in MC4R-/- mice [30].

### Abundant large sized follicles and abundant regressing follicles indicate a disturbed follicular dynamic in MC4R-/- mice

Differences between MC4R-/- and MC4R+/+ mice were noted in the number of small and large follicles. However the differences were more striking among antral follicles which are hypothalamic/pituitary gland-dependent and represent later stages of folliculogenesis. A substantially higher number of larger follicles was seen in ovaries of MC4R-/- mice rather than in MC4R+/+ mice, in particular in ovaries of mice with vaginal signs of estrus. In addition, the follicles were more frequently of cystic appearance and had a diameter greater than 400 μm. Cystic follicles are supposed to arise from anovulatory follicles, which continue to enlarge during the subsequent and following cycles [31]. They are suggested to result from an inadequate gonadotrophic stimulation of preovulatory follicles and are not to be involved in the subsequent ovarian cycles [32].

In contrast to MC4R+/+ mice, large-sized follicles in MC4R-/- mice frequently underwent atresia. Final stages of follicular atresia were indicated by collapsed zonae pellucidae. The wider range in their number in MC4R-/- mice supports the notion that cyclic follicular development and hypothalamic/pituitary-gonadal signalling is probably disturbed in MC4R-/- mice.

The ovarian changes in the MC4R-/- mice discussed here are reminiscent of the polycystic ovary syndrome (PCOS), which frequently comes along with obesity. PCOS is one of the most common causes of infertility in women of reproductive age in Western and industrialized countries [33]. The patients show increased androgen levels. Aspects of PCOS are well described for many other obese mouse models with a single gene defect [34], yet none of these models reveal high androgen serum levels.

In rodents, MC4R appears to be exclusively expressed in the brain, mainly in the hypothalamus [35,36], although expression in peripheral tissues has been suggested [35,37]. There is no direct evidence for hypothalamic neurons expressing GnRH and functional MC4R *in vivo*. However, MC4R mRNA is found in neurons of the paraventricular nucleus [35]. α-melanocyte stimulating hormone (α-MSH)-immunopositive nerve fibers are located close to GnRH producing neurons [38]. Additionally GnRH release is coupled to a functional MC4R in immortalized mouse GnRH neurons *in vitro *[39]. Furthermore, α-MSH stimulates luteinizing hormone (LH) [40] and preovulatory prolactin secretion *in vivo *[41]. All these reports suggest a direct modulation of the hypothalamic/pituitary-gonadal axis by MC4R signaling [42] and a neuroendocrine dysregulation in case of a partial or complete loss of MC4R function. On the other hand, a decrease in CL formation together with weight gain is suggestive of obesity-associated changes, such as hyperleptinemia followed by leptin resistance [43] or disturbed androgen levels with ovarian dysregulation [44,45].

### MC4R-deficient female mice develop interstitial gland cell hypertrophy

Interstitial gland cells appear to originate from the theca interna of regressing follicles [46]. As the ovary continues to lose follicles by regression there is an increase in interstitial gland cells. This process has been suggested for the human menopausal ovary [47]. In some species, including mice, interstitial gland cells form clusters separated by undifferentiated stromal cells [48]. Most notably, the cells synthesize C19 androgens [49]. When not aromatised to estrogens by granulosa cells, androgens can cause follicular regression. For MC4R-/- mice, large-sized antral follicles and cystic follicles are found. It is thus conceivable that the epithelioid-like interstitial gland cells in MC4R-/- mice synthesize more androgen than interstitial gland cells in MC4R+/+ mice. Normally, interstitial gland cells are fibroblast-like cells. LH stimulates them to produce androgens [48,49] and promotes shaping to large polyhedral epithelioid-like cells. Insulin, which is increased in MC4R-/- mice with late-onset obesity, belongs to the factors that augment LH sensitivity of interstitial gland cells [47]. Lipid droplets excessively accumulate in interstitial gland cells in mice with leptin-gene deficiency [50]. Lipid droplet accumulation is not noted in the ovary of the obese MC4R-deficient mice.

### MC4R-deficient female mice show a reduction in the number of CL

By counting CL in completely dissected ovaries, we determined an estimate for the number of eggs shed from the ovaries i.e. an estimate for the ovulation rate [51]. Corpora lutea from present and previous estrous cycles decreased in MC4R-/- mice between 3 to 5 months of age and were markedly reduced or absent in 6-month-old MC4R-/- mice. The reduction in CL points to a decrease in follicle rupture and CL formation for several estrous cycles. This finding is supported by the high proportion of MC4R-/- mice with vaginal signs of estrus (23 out of 30 mice in total). Cessation of cyclicity in B6 mice is followed by a period of vaginal cornification, which is morphological characterized by absence of CL, polyfollicular ovaries and, as the term implies, persistent vaginal stratification and cornification [30]. It is of note that the vaginal epithelium of these "persistently estrous mice" does not require estrogen for its constant cornification [30,52] and that the changes persist for longer the earlier cessation of cyclicity is established [30]. The age-depended decrease in ovulations explains the observed difficulties in obtaining offspring with older female MC4R-/- mice. Reduction of CL in both mice populations with differing strain background implies a general phenotype caused by a loss of MC4R function.

### Conclusion and Perspectives

The link between obesity and its effects on the reproductive system is not well understood. We here report for the first time a decrease in the number of CL in obese MC4R-/- mice, which progresses early in reproductive life and likely causes infertility. Although this study is a morphologic study, it is nevertheless important for understanding the MC4R system and its role in female reproduction. It will be of interest in future work to examine whether loss of weight by voluntary exercise restores ovarian cycles, as has been shown for erectile dysfunction in male MC4R-deficient mice [15]. Future hormone analysis of MC4R-/- mice will give insights into serum levels of gonadotropins and of sex steroids during reproductive aging. In conclusion, MC4R-/- mice appear to provide an important animal model to study the influence of the melanocortin-MC4R system in female reproduction.

## Competing interests

The authors declare that they have no competing interests.

## Authors' contributions

MS processed and evaluated the histological material, analysed the data and drafted a first manuscript. AS handled and genotyped the mice. CM helped in the laboratory. TS, KS and AR supervised the project and edited the manuscript. All authors read and approved the final manuscript.
